# Complete genome sequences of bacteriophages P12002L and P12002S, two lytic phages that infect a marine *Polaribacter* strain

**DOI:** 10.1186/s40793-015-0076-z

**Published:** 2015-10-19

**Authors:** Ilnam Kang, Hani Jang, Jang-Cheon Cho

**Affiliations:** Department of Biological Sciences, Inha University, Incheon, 402-751 Republic of Korea

**Keywords:** Bacteriophage, *Polaribacter*, *Bacteroidetes*, Genome, *Siphoviridae*

## Abstract

The bacterial genus *Polaribacter* is distributed widely in marine environments; however, there have been no reports of phages infecting *Polaribacter* strains. Here, we describe the isolation and genome sequencing of two lytic siphophages, P12002L and P12002S, that infect *Polaribacter* sp. strain IMCC12002. The two phages and host strain were isolated from coastal seawater of Korea. Complete genome sequences of the two phages were similar to each other and about 50 kb in length, with a G + C content of 28.9 %. The two genomes showed typical characteristics of phage genomes: a modular structure and high proportion of hypothetical proteins. The genome sequences have been deposited in GenBank under accession numbers KR136259 (P12002L) and KR136260 (P12002S).

**Electronic supplementary material:**

The online version of this article (doi:10.1186/s40793-015-0076-z) contains supplementary material, which is available to authorized users.

## Introduction

Viruses are the most numerous biological entities in the marine water column. The number of virus-like particles is known to exceed that of prokaryotes by approximately 10-fold on average [1]. Recent metagenomic studies on marine viruses have added an immense amount of novel sequence data to public databases [2]. However, the functional and phylogenetic assignment of these novel viral sequences is daunting, partly due to the shortage of available reference genomes [3]. Since most viruses in marine environments are believed to be bacteriophages [4], isolation and genome-based characterization of phages infecting widespread marine bacterial groups are important for a thorough understanding of marine viral diversity.

The genus *Polaribacter*, affiliated with the family *Flavobacteriaceae* of the phylum *Bacteroidetes*, currently contains 14 validly published species identified from marine environments. Several studies have shown that the genus is widely distributed in marine ecosystems, including polar seas and the North Sea [5–7], indicating that the genus *Polaribacter* may play important ecological roles and also suggesting the importance of any *Polaribacter* phages.

To our knowledge, however, no phages infecting the genus *Polaribacter* have been reported. In fact, phages infecting the family *Flavobacteriaceae*, to which *Polaribacter* belongs, have rarely been isolated, and currently only a limited number of bacterial groups within the *Flavobacteriaceae* are known to have phages infecting them [8]. This situation has posed a conundrum in virome studies of marine environments, since the family *Flavobacteriaceae* is one of the major bacterial assemblages found in the marine water column [9, 10].

Here, we report the genome sequences of two lytic bacteriophages, P12002L and P12002S, that infect strain IMCC12002, a marine bacterium phylogenetically assigned to the genus *Polaribacter*. The genome sequences of phages P12002L and P12002S represent the first addition of *Polaribacter* phage genomes to public sequence databases.

## Virus information

### Classification and features

P12002L and P12002S are lytic phages that infect the bacterial strain *Polaribacter* sp. IMCC12002. A coastal seawater sample collected off Incheon Harbor, located on the west coast of South Korea, was serially diluted with autoclaved seawater and spread onto marine agar plates (Difco). Strain IMCC12002 was established from a colony grown on a plate after two weeks of incubation at 20 °C in the dark by three rounds of plate streaking. Phylogenetic analyses based on 16S rRNA gene sequences placed strain IMCC12002 within the genus *Polaribacter* of the family *Flavobacteriaceae*. Sequence similarities of the 16S rRNA gene between IMCC12002 and type strains of the genus *Polaribacter*, calculated using EzTaxon-e server [11], ranged from 95.0 % to 98.4 %, with a maximum value found for *Polaribacter irgensii* 23-P^T^ [12]. Strain IMCC12002 was routinely grown in mR2A media.

Phages P12002L and P12002S were isolated from a surface seawater sample collected from the same station where the host strain IMCC12002 was isolated previously. An enrichment culture was performed to increase the concentration of phages putatively infecting strain IMCC12002 prior to plaque assay. Broth culture of IMCC12002 (20 ml) in the exponential phase was mixed with 380 ml of a 0.2-μm filtered seawater sample and 100 ml of 5× mR2A broth, and was further incubated at 20 °C for a week. During incubation, 10 ml of culture broth was withdrawn three times (at 2-day intervals) and vortexed for 5 min after the addition of 2 ml of chloroform. The chloroform was separated by centrifugation and the aqueous phase was recovered and stored at 4 °C for subsequent use in plaque assays. Plaque assays were performed using the double agar layer method. Bottom and top agar layers were prepared by the addition of Bacto Agar (BD Difco) to mR2A broth, at concentrations of 1.5 % and 0.7 % (w/v), respectively. Exponentially growing IMCC12002 (0.5 ml) and the chloroform-treated enrichment culture (0.5 ml; diluted if necessary) were added to 6 ml of molten (50 °C) top agar and poured onto bottom agar plates. After solidification of the top agar layer, plates were incubated at 20 °C for a week. Two plaques were selected from the assay plates, then were further purified through three cycles of picking, elution, dilution, and plaque assay, and were established and designated phages P12002L and P12002S. The two phages formed clear plaques on the host bacterial lawn after 1–2 days of incubation at 20 °C. The diameters of plaques were 1–2 mm for both phages, although the two phages seemed to show a difference in plaque size on the original assay plates.

**Fig. 1:**
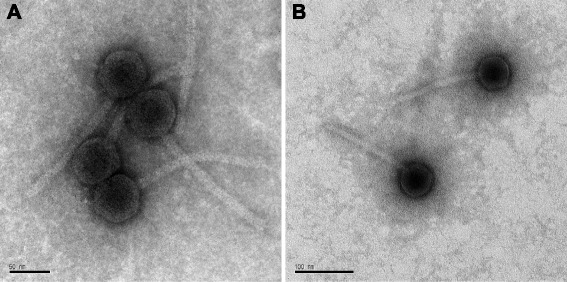
Transmission electron micrographs of *Polaribacter* phages P12002L (**a**) and P12002S (**b**). Scale bar, 50 nm in (**a**) and 100 nm in (**b**)

Morphological and genomic characterization were performed for classification of the two phages, P12002L and P12002S, using viral particles amplified and purified as described below (see Genome sequencing information). For morphological characterization, purified phage particles were adsorbed onto 200-mesh formvar and carbon-coated copper grids (Electron Microscopy Sciences), stained with uranyl acetate solution (2 %, w/v), and then examined by transmission electron microscope (CM200, Phillips). Both phages had isometric heads of about 55 nm in diameter and long non-contractile tails of about 150 nm in length (Fig. 1). For genomic characterization, nucleic acids were extracted from phage particles and treated with restriction enzymes. The genomic material of both phages were digested by enzymes such as *Nde* I and *Hha* I, showing that the two phages possessed dsDNA as their genomic material. Taken together, these results indicated the affiliation of these phages with the family *Siphoviridae* [13].

**Fig. 2:**
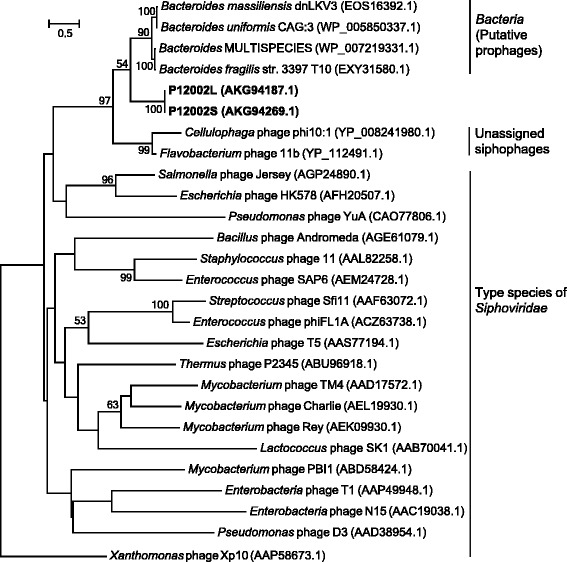
Maximum-likelihood phylogenetic tree based on TerL protein sequences indicating the position of P12002L and P12002S (shown in bold). All sequences, except for P12002L and P12002S, were obtained from GenBank. Sequences were aligned by MUSCLE [36]. Tree building was performed by RAxML [37], using the “-f a” method, PROTGAMMAJTT model, and MRE-based bootstopping criterion. Bootstrap replicate searches were performed 400 times, and bootstrap values (>50 %) are shown at branch nodes. *Xanthomonas* phage Xp10 was set as the root after tree building. Bar, 0.5 substitutions per amino acid position

Genes encoding TerL were predicted from the genomes of both phages and were used for inference of the phylogenetic positions of the phages. Although mosaicism is known to be rather prevalent in phage genomes [14] and the family *Siphoviridae* is underexplored with regard to marker (signature) genes [15], the terminase large subunit gene has been widely used as a marker gene in phage studies [16, 17]. TerL amino acid sequences of the two phages were identical and only distantly related to those of other phages representing the diverse genera of *Siphoviridae* [18] (Fig. 2). The most similar TerL proteins among prokaryotic isolates were found mainly in the genomes of *Bacteroides*, most of which were isolated from animals including humans. Among phage isolates included in the nr database of GenBank, the highest similarities were recorded for *Cellulophaga* phage phi10:1 [8] and *Flavobacterium* phage 11b [19], both of which were isolated from marine environments and that are not yet assigned to any known genera of *Siphoviridae*. Taken together, TerL-based phylogeny suggested that P12002L and P12002S, the siphoviruses isolated in this study, occupied a phylogenetic position distinct from previously established genera of the family *Siphoviridae*.

Information on the isolation, classification, and general features of the two phages are presented in Table 1.

**Table 1:** Classification and general features of phages P12002L and P12002S according to the MIGS recommendations [39]

MIGS ID	Property	Term	Evidence code^a^
	Classification	Domain: unassigned (ds DNA viruses)	
		Phylum: unassigned	
		Class: unassigned	
		Order *Caudovirales*	TAS [13]
		Family *Siphoviridae*	TAS [13]
		Genus: unassigned	
		Species: unassigned	
		Strains: P12002L, P12002S	
	Particle shape	Isometric capsid with a long non-contractile tail	IDA
MIGS-6	Habitat	Marine water column	IDA
MIGS-15	Biotic relationship	Intracellular parasite of *Polaribacter* strain IMCC12002	IDA
MIGS-14	Pathogenicity	Non-pathogenic	NAS
MIGS-4	Geographic location	The Yellow Sea, Incheon, South Korea	IDA
MIGS-5	Sample collection	March 16, 2010	IDA
MIGS-4.1	Latitude	37° 29′ 51′′ N	IDA
MIGS-4.2	Longitude	126° 38′ 26′′ E	IDA
MIGS-4.3	Depth	0.3 m	IDA
MIGS-4.4	Altitude	-	-

^a^Evidence codes - *IDA* Inferred from Direct Assay, *TAS* Traceable Author Statement, *NAS* Non-traceable Author Statement. These evidence codes are from the Gene Ontology project [40]

## Genome sequencing information

### Genome project history

Genome sequencing of the two phages, P12002L and P12002S, was performed as a part of a research project that aimed to increase the number of marine phage genomes in public databases, which is expected to lay the foundation for a more thorough understanding of the (meta)genomic diversity in marine environments, as exemplified by studies on pelagiphages and SAR116 phages [20–22]. Previously, we reported the genome sequences of several other marine phages infecting diverse host bacteria [23–25], which have been utilized for marine virus studies [26, 27].

Genome sequencing of P12002L and P12002S was performed using an Illumina HiSeq platform using a 100-bp paired-end library generated from DNA samples extracted from concentrated and purified phage particles. Assembly by SOAPdenovo [28], followed by gap closing by PCR, resulted in a single circular contig for each phage. Gene calling and annotation were carried out mainly using the RAST server [29]. The complete genome sequences and annotation information of both phages were submitted to GenBank, with accession numbers KR136259 (P12002L) and KR136260 (P12002S). Information on these genome sequencing projects is available through the Genomes OnLine Database under project numbers Gp0115710 (P12002L) and Gp0115711 (P12002S), and a summary of these projects is shown in Table 2.

**Table 2:** Project information

MIGS ID	Property	Term
MIGS-31	Finishing quality	Finished
	Number of contigs	1
MIGS-28	Libraries used	One paired-end Illumina library (per each phage)
MIGS-29	Sequencing platforms	Illumina HiSeq
MIGS-31.2	Fold coverage	P12002L, ~29,000×; P12002S, ~33,000×
MIGS-30	Assemblers	SOAPdenovo
MIGS-32	Gene calling method	RAST gene caller
	GenBank ID	P12002L, KR136259; P12002S, KR136260
	GenBank Date of Release	May 16, 2015
	GOLD ID	P12002L, Gp0115710; P12002S, Gp0115711
MIGS-13	Source material identifier^a^	P12002L, P12002S
	Project relevance	Diversity of marine bacteriophage

^a^Viruses have not been deposited yet

### Growth conditions and genomic DNA preparation

Phages P12002L and P12002S, isolated as described above, were subsequently amplified in liquid culture using bacterial strain IMCC12002 as a host. IMCC12002 was grown at 20 °C with shaking at 100 rpm in 200 ml of mR2A broth until the late lag or early exponential phase before concentrated phage particles were inoculated. Host bacteria were clearly lysed within 24 h of phage inoculation. After lysis, extracellular nucleic acids were digested by the addition of DNase I (Sigma) and RNase A (Qiagen) (each 1 μg ml^−1^), followed by incubation at 37 °C for 1 h. Subsequently, sodium chloride was added at a final concentration of 0.5 M and lysed cultures were stored in iced water for 1 h before removal of cellular debris by centrifugation (12,000 × *g*, 30 min). PEG 8000 (Sigma) was added to recovered supernatants (10 %, w/v) and the mixture was stored at 4 °C overnight. Phage particles were pelleted by centrifugation (12,000 × *g*, 30 min). After discarding supernatants, pellets were resuspended in SM buffer. PEG was removed by treatment with an equal volume of chloroform, followed by phase separation by centrifugation (2,500 × *g*, 1 h). Phage particles in the aqueous phase were further purified by equilibrium gradient ultracentrifugation (SW 55 Ti Rotor, 40,000 rpm, 24 h, 4 °C) after addition of CsCl (Sigma; 0.75 g per ml of phage concentrates). Phage bands formed in ultracentrifuge tubes were withdrawn with syringes. After removal of CsCl by repeated ultrafiltration, phage samples were used for DNA extraction with a DNeasy Blood & Tissue Kit (Qiagen).

### Genome sequencing and assembly

Extracted DNA samples were used for generation of shotgun libraries (one library for each phage), which were sequenced (2× 100 bp) by Macrogen, Inc. (Korea) using an Illumina HiSeq platform. In total, about 7.1 and 8.3 million pairs of reads were obtained for P12002L and P12002S, respectively. Raw reads were assembled by SOAPdenovo after being partitioned into subsets composed of 50,000–200,000 paired reads, with a *k*-mer value of 85. Partition seemed to be necessary since the assembly of non-partitioned whole sequence data resulted in a greater number of shorter contigs when compared to the assembly results obtained from partitioned subsets. For both phages, several partitioned subsets of different sizes were assembled separately, which produced nearly the same single contig for all subsets. Primers were designed from the ends of contigs with an outward orientation and used in PCR, with the genomic DNA of phages used as templates. The sequences of PCR products were determined by Sanger sequencing and were used to close the circular contigs for both phages, eventually resulting in a single contig for each phage. Coverages, roughly estimated from the sequencing statistics and final contig sizes, were about 29,000 and 33,000 for P12002L and P12002S, respectively (Table 2).

### Genome annotation

Gene calling and annotation of the complete phage genomes were performed by the RAST server with FIGfam version of release 70. Gene caller was set to ‘RAST’, and ‘Fix frameshifts’ and ‘Backfill gaps’ were selected. Functional annotation of protein coding genes was improved by RPS-BLAST searches against the Conserved Domain Database (CDD), available through the web interface [30], and HMMER searches against UniProtKB [31], the latter of which was also used to predict signal peptides and transmembrane helices by the Phobius program [32]. BLASTp searches against NCBI nr database were also performed. To calculate COG statistics from RPS-BLAST results, two files downloaded from the NCBI FTP server were used: “cognames2003–2014.tab” and “fun2003–2014.tab” [33]. Coding densities of the genomes were calculated using the “Create Intergenic Features” function of Artemis [34], based on Genbank files generated by the RAST server.

## Genome properties

The complete genomes of two phages were assembled into single circular contigs of 48,689 bp (P12002L) and 49,847 bp (P12002S) in length. Considering that the genomes of tailed dsDNA phages are known to be linear, this circular assembly suggests that the genomes were circularly permuted or terminally redundant [35]. The G + C contents of the two genomes were 28.9 %. The number of protein coding genes predicted in the two phage genomes were 82 (P12002L) and 86 (P12002S). No RNA genes or pseudogenes were predicted. Among the protein coding genes, only 16 and 14 genes in P12002L and P12002S, respectively, were assigned putative functions, while the other genes were annotated as hypothetical proteins. The annotation summaries for the two genomes are presented in Table 3 and detailed annotation information is shown in Additional file 1: Tables S1 and S2. Distributions of the protein functions among COG functional categories are shown in Table 4.

**Table 3:** Genome statistics

Attribute	P12002L		P12002S	
	Value	% of Total^a^	Value	% of Total^a^
Genome size (bp)	48,689	100.00	49,847	100.00
DNA coding (bp)	43,666	89.68	44,538	89.35
DNA G + C (bp)	14,088	28.93	14,422	28.93
DNA scaffolds	1	100.00	1	100.00
Total genes	82	100.00	86	100.00
Protein coding genes	82	100.00	86	100.00
RNA genes	0	0.00	0	0.00
Pseudo genes	0	0.00	0	0.00
Genes in internal clusters	0	0.00	0	0.00
Genes with function prediction	16	19.51	14	16.28
Genes assigned to COGs	9	10.98	9	10.47
Genes with Pfam domains	18	21.95	15	17.44
Genes with signal peptides	1	1.22	0	0.00
Genes with transmembrane helices	4	4.89	8	9.30
CRISPR repeats	0	0.00	0	0.00

^a^The total is based on the size of the genome in base pairs or the total number of protein-coding genes in the annotated genome

**Table 4:** Number of genes associated with general COG functional categories

Code	P12002L		P12002S		Description
	Value	% age	Value	% age	
J	0	0	0	0	Translation, ribosomal structure and biogenesis
A	0	0	0	0	RNA processing and modification
K	0	0	0	0	Transcription
L	5	6.10	6	6.98	Replication, recombination and repair
B	0	0	0	0	Chromatin structure and dynamics
D	1	1.22	1	1.16	Cell cycle control, cell division, chromosome partitioning
V	1	1.22	2	2.33	Defense mechanisms
T	0	0	0	0	Signal transduction mechanisms
M	0	0	0	0	Cell wall/membrane biogenesis
N	0	0	0	0	Cell motility
U	0	0	0	0	Intracellular trafficking and secretion
O	0	0	0	0	Posttranslational modification, protein turnover, chaperones
C	0	0	0	0	Energy production and conversion
G	0	0	0	0	Carbohydrate transport and metabolism
E	0	0	0	0	Amino acid transport and metabolism
F	0	0	0	0	Nucleotide transport and metabolism
H	0	0	0	0	Coenzyme transport and metabolism
I	0	0	0	0	Lipid transport and metabolism
P	0	0	0	0	Inorganic ion transport and metabolism
Q	0	0	0	0	Secondary metabolites biosynthesis, transport and catabolism
R	4	4.88	3	3.49	General function prediction only
S	0	0	1	1.16	Function unknown
X	1	1.22	1	1.16	Mobilome: prophages, transposons
-	73	89.02	77	89.53	Not in COGs

**Fig. 3:**
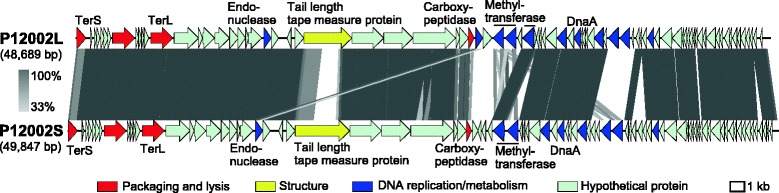
Genome maps of P12002L and P12002S. Protein coding genes are represented by arrows and their functional categories are coded in color as follows: Red, Packaging and lysis; Yellow, Structure; Blue, DNA metabolism/replication. Similarites between the two genomes were calculated based on tBLASTx and indicated in grey according to the scale at the left. This figure was drawn by Easyfig [38]

The two phage genomes showed a modular structure, were syntenic over the whole genomes, and shared many genes, including terminase, carboxypeptidase, tail length tape measure protein, methyltransferase, replication initiation protein DnaA, and endonucleases (Fig. 3).

## Conclusion

P12002L and P12002S, two lytic siphophages isolated from coastal seawater of Korea, infect *Polaribacter* sp. strain IMCC12002, and therefore represent the first phages of the genus *Polaribacter*, a taxonomic group found widely in the marine water column. Complete genome sequences of the two phages were obtained by Illumina sequencing, and were annotated by the RAST server and searches against various databases. The two phages showed synteny over the whole length (≈50 kb) of their genomes and shared many genes. A phylogenetic analysis of TerL protein sequences suggested that the two phages might constitute a novel genus-level group of *Siphoviridae*.

## Additional file

Additional file 1: Tables S1 and S2. Detailed annotation information of the two phage genomes. (PDF 80 kb)

## Abbreviation

TerL: Terminase large subunit
mR2A: R2A broth dissolved in diluted aged seawater (seawater:distilled water = 4:1)
