# Supplementary material for: Complete genome sequences of bacteriophages P12002L and P12002S, two lytic phages that infect a marine Polaribacter strain
Source: Stand Genomic Sci. 2015 Oct 19;10:82. doi: 10.1186/s40793-015-0076-z (PMC4615864; doi:10.1186/s40793-015-0076-z)
Supplement: Additional file 1: Tables S1 and S2. — Detailed annotation information of the two phage genomes. (PDF 80 kb) [file 40793_2015_76_MOESM1_ESM.pdf]

Table S1. Annotation of ORFs predicted in the P12002L genome

ORF	Position (strand)	Length (aa)	Predicted function	Best BLASTp hit from NCBI nr (organism, accession number, bitscore)	Pfam, COG, signal peptide (SP), and transmembrane helix (TM)
1	88-726 (+)	212	Terminase small subunit	hypothetical protein (*Mesoflavibacter zeaxanthinifaciens*, WP_027880403, 53.9)	PF03592 (Terminase small subunit)
2	1035-1154 (+)	39		lipoyl synthase (*Buchnera aphidicola*, WP_025368908, 33.1)	
3	1289-1474 (+)	61		hypothetical protein (*Sulfuricurvum kujiense*, WP_013449842, 37)	
4	1477-1692 (+)	71		hypothetical protein (*Reticulomyxa filosa*, ETO20733, 38.1)	
5	1757-1984 (+)	75		MULTISPECIES: hypothetical protein (Arcobacter, WP_026807899, 36.2)	
6	1965-2288 (+)	107		hypothetical protein (*Clostridium symbiosum*, WP_003502009, 37)	
7	2257-2454 (+)	65		hypothetical protein (*Salmonella enterica*, WP_010892316, 32.7)	
8	2580-4142 (+)	520	Probable A1 protein	A1-like protein (*Cellulophaga* phage phi4:1 , YP_008240608, 316.6)	
9	4201-4401 (+)	66		hypothetical protein (*Knoellia aerolata* DSM 18566, KGN40035, 38.5)	
10	4402-4596 (+)	64		hypothetical protein (*Flavobacterium* phage 11b, YP_112537, 77)	PF11753 (Protein of unknown function)
11	4609-4782 (+)	57		predicted protein (*Verticillium alfalfae* VaMs.102, XP_003004765, 32.7)	
12	4794-5108 (+)	104		hypothetical protein (*Elizabethkingia meningoseptica*, KGT09513, 56.2)	
13	5260-6801 (+)	513	Terminase large subunit	phage portal protein (*Bacteroides cellulosilyticus*, WP_007219331, 444.1)	PF04466 (Phage terminase large subunit), PF03237 (Terminase-like family)
14	6898-8610 (+)	570		hypothetical protein (*Robiginitalea biformata*, WP_015755469, 428.7)	
15	8588-8890 (+)	100		hypothetical protein (*Methylobacterium nodulans*, WP_015929254, 39.7)	
16	8902-9720 (+)	272		hypothetical protein (*Robiginitalea biformata*, WP_041327287, 98.2)	
17	9755-10777 (+)	340		hypothetical protein (*Robiginitalea biformata*, WP_015755466, 236.1)	
18	10779-11414 (+)	211		hypothetical protein (*Robiginitalea biformata*, WP_015755465, 135.2)	
19	11368-11901 (+)	177		hypothetical protein (*Flavobacterium* sp. CF136, WP_007804420, 102.8)	
20	11903-12106 (+)	67			
21	12109-13032 (+)	307		hypothetical protein (*Chryseobacterium* sp. YR480, WP_047425770, 45.4)	
22	13184-13696 (+)	170	HNH endonuclease	HNH endonuclease (*Gramella forsetii*, WP_011710355, 122.9)	PF07463 (NUMOD4 motif), PF13392 (HNH endonuclease), PF00847 (AP2 domain)
23	13709-14215 (+)	168		hypothetical protein (*Robiginitalea biformata*, WP_015755461, 49.3)	COG1713
24	15093-14812 (-)	93		protein methyltransferase FrzF (*Myxococcus xanthus*, WP_011554141, 35)	
25	15356-15982 (+)	208		hypothetical protein (*Chryseobacterium* sp. OV259, WP_034758591, 44.7)	
26	15972-19346 (+)	1124	Tail length tape measure domain	tape measure domain (uncultured Mediterranean phage uvMED, BAQ84149, 302.7)	PF02463 (RecF/RecN/SMC N terminal domain), PF05483 (Synaptonemal complex protein 1), COG3941, COG1196, COG5022
27	19349-21577 (+)	742		structural protein (*Cellulophaga* phage phi19:1, YP_008241790, 263.5)	
28	21577-23616 (+)	679		structural protein (*Cellulophaga* phage phi19:1, YP_008241791, 179.5)	
29	23617-26610 (+)	997		hypothetical protein (*Mesorhizobium plurifarium*, WP_041005494, 165.3)	
30	26628-26915 (+)	95		hypothetical protein (*Kordia algicida*, WP_007093059, 49.3)	2 TM

31	26905-27528 (+)	207		hypothetical protein (*Genlisea aurea*, EPS66458, 38.5)	TM
32	27531-27917 (+)	128	carboxypeptidase	hypothetical protein (*Bizionia argentinensis*, WP_040287984, 110.2)	PF13539 (D-alanyl-D-alanine carboxypeptidase)
33	28031-28516 (+)	161	HNH endonuclease	HNH endonuclease (*Gramella forsetii*, WP_011710355, 124.8)	PF07463 (NUMOD4 motif), PF13392 (HNH endonuclease), PF00847 (AP2 domain)
34	28524-29165 (+)	213		hypothetical protein (*Flavobacterium rivuli*, WP_020212237, 100.1)	SP
35	29973-29248 (-)	241	Adenine-specific methyltransferase	hypothetical protein (*Acinetobacter lwoffii*, WP_005103760, 281.6)	PF01555 (DNA methylase), COG0863, COG2189, COG2521
36	30848-30078 (-)	256	Type III restriction-modification system methylation subunit	DNA methylase (*Cellulophaga* phage phi12:1, YP_008240985, 316.2)	PF01555 (DNA methylase), COG0863, COG2189
37	31255-30965 (-)	96		hypothetical protein (*Phytophthora parasitica* P1569, ETI55211, 37.4)	
38	32126-31326 (-)	266	Adenine-specific methyltransferase	DNA methylase (*Vibrio* phage pYD38-B, YP_008130270, 242.7)	PF01555 ((DNA methylase), COG0863, COG2189
39	32098-32271 (+)	57			
40	32455-32279 (-)	58		hypothetical protein (*Pseudocercospora fijiensis* CIRAD86, XP_007923413, 35.8)	
41	32638-32486 (-)	50		hypothetical protein (*Perkinsela* sp. CCAP 1560/4, KNH05471, 35.8)	
42	32917-32693 (-)	74		Hermansky-Pudlak syndrome 6 protein, partial (*Nestor notabilis*, XP_010009980, 34.7)	
43	33700-32933 (-)	255		hypothetical protein (*Cellulophaga* phage phi18:3, YP_008241198, 85.5)	
44	34375-33707 (-)	222	Replication protein	DNA-binding protein (*Campylobacter* sp. FOBRC14, WP_009651041, 120.9)	PF13730 (Helix-turn-helix domain)
45	34893-34489 (-)	134		hypothetical protein (*Polaribacter* sp. Hel_I_88, WP_026776786, 100.9)	PF01503 (Phosphoribosyl-ATP pyrophosphohydrolase)
46	35382-34903 (-)	159	chromosome replication initiation protein DnaA	chromosomal replication initiator protein DnaA (*Lachnospiraceae* bacterium ND2006, WP_035638943, 60.8)	PF08299 (Bacterial dnaA protein helix-turn-helix), COG0593
47	35503-35372 (-)	43			
48	35700-35470 (-)	76		hypothetical protein (*Pedobacter* sp. BMA, WP_047801173, 40)	
49	35863-35675 (-)	62			
50	36309-35863 (-)	148		hypothetical protein (*Eremothecium cymbalariae* DBVPG#7215, XP_003645407, 37.7)	
51	36973-36311 (-)	220	NinG recombination protein	hypothetical protein, partial (*Proteobacteria* bacterium JGI 0000113-P07, WP_036929766, 77)	PF05766 (Bacteriophage Lambda NinG protein)
52	37199-36951 (-)	82		unnamed protein product (*Sordaria macrospora* k-hell, CCC10760, 37)	
53	38015-37305 (-)	236	DNA modification protein	gp178 (uncultured Mediterranean phage uvMED, BAR35039, 183)	
54	38816-38118 (-)	232	DNA methylase	DNA methylase (*Cellulophaga* phage phi12:1, YP_008241004, 338.6)	PF02086 (D12 class N6 adenine-specific DNA methyltransferase), PF01555 (DNA methylase), COG0338
55	39099-38950 (-)	49			
56	39480-39253 (-)	75		hypothetical protein (*Cellulophaga* phage phi19:1, YP_008241803, 53.5)	
57	39664-39530 (-)	44			

58	39995-39708 (-)	95		MULTISPECIES: hypothetical protein (*Pseudomonas putida* group, WP_019751570, 36.6)	
59	40502-40062 (-)	146	HNH endonuclease	HNH endonuclease (*Streptococcus constellatus*, WP_048800727, 131.3)	PF01844 (HNH endonuclease), COG1403
60	40896-40567 (-)	109		UDP-N-acetylglucosamine 2-epimerase (*Paulinella chromatophora*, YP_002049387, 39.3)	
61	41779-41006 (-)	257		hypothetical protein (*Flavobacterium* phage FCL-2, YP_009140550, 106.3)	TM
62	42449-41784 (-)	221		hypothetical protein, partial (*Candidatus* Caldatribacteirum californiense, WP_017890182, 77.4)	PF04098 (Rad52/22 family double-strand break repair protein)
63	42600-42463 (-)	45		hypothetical protein (*Rhizopus microsporus*, CEI91090, 36.2)	PF09889 (Uncharacterized protein containing a Zn-ribbon), COG4068
64	42796-42587 (-)	69		hypothetical protein (*Corynebacterium kroppenstedtii* DSM 44385, ACR18580, 34.7)	
65	42970-42827 (-)	47		MerR family transcriptional regulator (*Clostridium tetanomorphum*, WP_035151379, 35)	
66	43183-42974 (-)	69		sam/hd domain protein, putative (*Entamoeba invadens* IP1, XP_004260964, 33.9)	
67	43383-43246 (-)	45			
68	43541-43380 (-)	53			
69	43931-43542 (-)	129		hypothetical protein (uncultured Mediterranean phage uvMED, BAQ92845, 62)	
70	44158-43946 (-)	70			
71	44821-44162 (-)	219		hypothetical protein (*Elizabethkingia anophelis*, WP_024566412, 62.4)	
72	45125-44835 (-)	96		hypothetical protein (*Enterococcus caccae* ATCC BAA-1240, EOL45101, 37.4)	
73	45339-45130 (-)	69		polysaccharide biosynthesis protein CapD (*Listeria cornellensis* FSL F6-969, EUJ25228, 36.2)	
74	45774-45442 (-)	110			
75	45957-45787 (-)	56		twin-arginine translocation protein subunit TatB (*Desulfovibrio salexigens*, WP_015851433, 33.5)	
76	46332-46027 (-)	101		hypothetical protein (*Calothrix* sp. PCC 7103, WP_019495525, 37.4)	
77	46573-46340 (-)	77		hypothetical protein (*Zavarzinella formosa*, WP_029630428, 60.8)	
78	46914-46585 (-)	109		UNKNOWN (*Stylonychia lemnae*, CDW79434, 37)	TM
79	47177-46947 (-)	76			
80	47427-47239 (-)	62			
81	47706-47485 (-)	73		hypothetical protein (*Listeria* phage LP-037, YP_008240523, 45.8)	
82	48107-47961 (-)	48			

COG and Pfam were searched by RPS-BLAST against CDD. Additional Pfam search was performed by hmmscan during HMMER analysis. SP and TM were predicted by the Phobius program as implemented at the HMMER web server.

Table S2. Annotation of ORFs predicted in the P12002S genome

ORF	Position (strand)	Length (aa)	Predicted function	Best BLASTp hit from NCBI nr (organism, accession number, bitscore)	Pfam, COG, signal peptide (SP), and transmembrane helix (TM)
1	54-710 (+)	218	Terminase small subunit	phage small terminase (*Gramella forsetii*, WP_011710310, 55.5)	
2	1019-1138 (+)	39		lipoyl synthase (*Buchnera aphidicola*, WP_025368908, 33.1)	
3	1273-1458 (+)	61		hypothetical protein (*Sulfuricurvum kujiense*, WP_013449842, 37)	
4	1461-1676 (+)	71		hypothetical protein (*Reticulomyxa filosa*, ETO20733, 38.1)	
5	1741-1968 (+)	75		MULTISPECIES: hypothetical protein (*Arcobacter*, WP_026807899, 36.2)	
6	1949-2272 (+)	107		hypothetical protein (*Clostridium symbiosum*, WP_003502009, 37)	
7	2241-2438 (+)	65		hypothetical protein (uncultured marine thaumarchaeote KM3_78_A04, AIF17572, 33.1)	
8	2564-4126 (+)	520	Probable A1 protein	A1-like protein (*Cellulophaga* phage phi4:1, YP_008240608, 316.6)	
9	4185-4385 (+)	66		hypothetical protein (*Knoellia aerolata* DSM 18566, KGN40035, 38.5)	
10	4386-4580 (+)	64		hypothetical protein (*Flavobacterium* phage 11b, YP_112537, 77)	PF11753 (Protein of unknown function)
11	4593-4766 (+)	57		predicted protein (*Verticillium alfalfae* VaMs.102, XP_003004765, 32.7)	
12	4778-5092 (+)	104		hypothetical protein (*Elizabethkingia meningoseptica*, KGT09513, 56.2)	
13	5244-6785 (+)	513	Terminase large subunit	phage portal protein (*Bacteroides cellulosilyticus*, WP_007219331, 444.1)	PF04466 (Phage terminase large subunit), PF03237 (Terminase-like family)
14	6882-8594 (+)	570		hypothetical protein (*Robiginitalea biformata*, WP_015755469, 428.7)	
15	8572-8874 (+)	100		hypothetical protein (*Methylobacterium nodulans*, WP_015929254, 39.7)	
16	8886-9704 (+)	272		hypothetical protein (*Robiginitalea biformata*, WP_041327287, 98.2)	
17	9739-10761 (+)	340		hypothetical protein (*Robiginitalea biformata*, WP_015755466, 236.1)	
18	10763-11398 (+)	211		hypothetical protein (*Robiginitalea biformata*, WP_015755465, 135.2)	
19	11352-11885 (+)	177		hypothetical protein (*Flavobacterium* sp. CF136, WP_007804420, 102.8)	
20	11887-12090 (+)	67			
21	12093-13016 (+)	307		hypothetical protein (*Chryseobacterium* sp. YR480, WP_047425770, 45.4)	
22	13168-13680 (+)	170	HNH homing endonuclease	HNH endonuclease (*Gramella forsetii*, WP_011710355, 122.9)	PF07463 (NUMOD4 motif), PF13392 (HNH endonuclease), PF00847 (AP2 domain)
23	13693-14199 (+)	168		hypothetical protein (*Robiginitalea biformata*, WP_015755461, 49.3)	COG1713
24	15077-14796 (-)	93		protein methyltransferase FrzF (*Myxococcus xanthus*, WP_011554141, 35)	
25	15340-15966 (+)	208		hypothetical protein (*Chryseobacterium* sp. OV259, WP_034758591, 44.7)	
26	15956-19777 (+)	1273	Tail length tape measure domain	tape measure domain (uncultured Mediterranean phage uvMED, BAQ84149, 308.9)	PF05483 (Synaptonemal complex protein 1), COG3941, COG1196, COG0419, COG4372, COG0610, 2 TM
27	19780-22008 (+)	742		structural protein (*Cellulophaga* phage phi19:1 , YP_008241790, 266.2)	
28	22008-24047 (+)	679		structural protein (*Cellulophaga* phage phi19:1 , YP_008241791, 179.5)	
29	24048-27041 (+)	997		hypothetical protein (*Mesorhizobium plurifarium*, WP_041005494, 165.3)	
30	27059-27346 (+)	95		hypothetical protein (*Kordia algicida*, WP_007093059, 48.9)	2 TM

31	27336-27959 (+)	207		hypothetical protein (*Genlisea aurea*, EPS66458, 38.1)	TM
32	27962-28318 (+)	118	Carboxypeptidase	hypothetical protein (*Bizionia argentinensis*, WP_040287984, 157.5)	PF13539 (D-alanyl-D-alanine carboxypeptidase), TM
33	28366-28776 (+)	136		hypothetical protein (*Flavobacterium limnosediminis*, WP_023579384, 49.3)	TM
34	28989-28753 (-)	78		hypothetical protein (*Flavobacterium psychrophilum*, WP_044049352, 68.6)	
35	29306-29037 (-)	89		unnamed protein product (*Mus musculus*, BAE35476, 37.7)	
36	29758-29453 (-)	101		(protein-PII) uridylyltransferase (*Azotobacter chroococcum*, WP_039802626, 38.9)	
37	30622-29822 (-)	266	DNA methylase	DNA methylase (*Vibrio* phage pYD38-B, YP_008130270, 242.7)	PF01555 (DNA methylase), COG0863, COG2189
38	31562-30798 (-)	254	DNA methylase	DNA methylase (*Cellulophaga* phage phi12:1, YP_008240985, 305.4)	PF01555 (DNA methylase), COG0863, COG2189
39	31865-31689 (-)	58		hypothetical protein (*Pseudocercospora fijiensis* CIRAD86, XP_007923413, 35.8)	
40	32048-31896 (-)	50		hypothetical protein (*Perkinsela* sp. CCAP 1560/4, KNH05471, 35.8)	
41	32327-32103 (-)	74		Hermansky-Pudlak syndrome 6 protein, partial (*Nestor notabilis*, XP_010009980, 34.7)	
42	33110-32343 (-)	255		hypothetical protein (*Cellulophaga* phage phi18:3, YP_008241198, 85.5)	
43	33785-33117 (-)	222	Replication protein	DNA-binding protein (*Campylobacter* sp. FOBRC14, WP_009651041, 120.9)	PF13730 (Helix-turn-helix domain)
44	34303-33899 (-)	134		hypothetical protein (*Polaribacter* sp. Hel_I_88, WP_026776786, 100.9)	PF01503 (Phosphoribosyl-ATP pyrophosphohydrolase)
45	34792-34313 (-)	159	Chromosomal replication initiator protein DnaA	chromosomal replication initiator protein DnaA (*Lachnospiraceae bacterium* ND2006, WP_035638943, 60.8)	PF08299 (Bacterial dnaA protein helix-turn-helix), COG0593
46	35110-34880 (-)	76		hypothetical protein (*Pedobacter* sp. BMA, WP_047801173, 40)	
47	35273-35085 (-)	62			
48	35719-35273 (-)	148		hypothetical protein (*Eremothecium cymbalariae* DBVPG#7215, XP_003645407, 37.7)	
49	36383-35721 (-)	220	NinG recombination protein	hypothetical protein, partial (Proteobacteria bacterium JGI 0000113-P07, WP_036929766, 77)	PF05766 (Bacteriophage Lambda NinG protein)
50	36609-36361 (-)	82		unnamed protein product (*Sordaria macrospora* k-hell, CCC10760, 37)	
51	36981-36676 (-)	101		hypothetical protein (*Chromobacterium violaceum*, WP_043617948, 35.8)	
52	37294-37043 (-)	83		AsnC family transcriptional regulator (*Selenomonas* sp. oral taxon 478, WP_050343091, 33.9)	
53	38053-37358 (-)	231	DNA methylase	Phage DNA modification methylase (*Microgenomates* bacterium GW2011_GWA2_46_16, KKU25463, 197.2)	PF01555 (DNA methylase), COG0863, COG2521
54	38896-38147 (-)	249	DNA methylase	DNA methylase (*Geobacillus* phage GBK2, YP_009010520, 261.2)	PF01555 (DNA methylase), COG0863
55	39300-39073 (-)	75		hypothetical protein (*Cellulophaga* phage phi19:1, YP_008241803, 53.5)	
56	39484-39350 (-)	44			

57	39823-39539 (-)	94		hypothetical protein (*Saccharospirillum* impatiens, WP_028669711, 37.7)	
58	40262-39969 (-)	97		ABC transporter ATP-binding protein (*Carnobacterium inhibens*, WP_034537685, 36.6)	
59	40566-40318 (-)	82		cobalamin synthesis protein (*Theileria annulata* strain Ankara , XP_955279, 35.4)	
60	40908-40621 (-)	95		MULTISPECIES: hypothetical protein (*Pseudomonas putida* group, WP_019751570, 36.6)	
61	41415-40975 (-)	146	HNH endonuclease	HNH endonuclease (*Streptococcus constellatus*, WP_048800727, 131.3)	PF01844 (HNH endonuclease), COG1403
62	41809-41480 (-)	109		UDP-N-acetylglucosamine 2-epimerase (*Paulinella chromatophora*, YP_002049387, 39.3)	
63	42692-41919 (-)	257		hypothetical protein (*Flavobacterium* phage FCL-2, YP_009140550, 106.3)	TM
64	43362-42697 (-)	221		hypothetical protein, partial (*Candidatus* Caldatribacteirum californiense, WP_017890182, 77.4)	
65	43513-43376 (-)	45		hypothetical protein (*Rhizopus microsporus*, CEI91090, 36.2)	PF09889 (Uncharacterized protein containing a Zn-ribbon), COG4068
66	43709-43500 (-)	69		hypothetical protein (*Corynebacterium kroppenstedtii* DSM 44385, ACR18580, 34.7)	
67	43883-43740 (-)	47		MerR family transcriptional regulator (*Clostridium tetanomorphum*, WP_035151379, 35)	
68	44096-43887 (-)	69		sam/hd domain protein, putative (*Entamoeba invadens* IP1, XP_004260964, 33.9)	
69	44296-44159 (-)	45			
70	44454-44293 (-)	53			
71	44844-44455 (-)	129		hypothetical protein (uncultured Mediterranean phage uvMED, BAQ92845, 61.6)	
72	45071-44859 (-)	70			
73	45734-45075 (-)	219		hypothetical protein (*Elizabethkingia anophelis*, WP_024566412, 62.4)	
74	46038-45748 (-)	96		hypothetical protein (*Enterococcus caccae* ATCC BAA-1240, EOL45101, 37.4)	
75	46252-46043 (-)	69		polysaccharide biosynthesis protein CapD (*Listeria cornellensis* FSL F6-969, EUJ25228, 36.2)	
76	46687-46355 (-)	110			
77	46870-46700 (-)	56		twin-arginine translocation protein subunit TatB (*Desulfovibrio salexigens*, WP_015851433, 33.5)	
78	47245-46940 (-)	101		hypothetical protein (*Calothrix* sp. PCC 7103, WP_019495525, 37.4)	
79	47486-47253 (-)	77		hypothetical protein (*Zavarzinella formosa*, WP_029630428, 60.8)	
80	47827-47498 (-)	109		UNKNOWN (*Stylonychia lemnae*, CDW79434, 37)	
81	48090-47860 (-)	76			TM
82	48340-48152 (-)	62			

83	48697-48464 (-)	77		myelin transcription factor 1-like protein isoform X6 (*Esox lucius*, XP_010893337, 37.7)	
84	48976-48755 (-)	73		hypothetical protein (*Listeria* phage LP-037, YP_008240523, 45.8)	
85	49134-49021 (-)	37			TM
86	49377-49231 (-)	48			

COG and Pfam were searched by RPS-BLAST against CDD. Additional Pfam search was performed by hmmscan during HMMER analysis. SP and TM were predicted by the Phobius program as implemented at the HMMER web server.
